# Hospital-based community eye health programme: A model for elimination of avoidable blindness on a sustainable basis

**Published:** 2017

**Authors:** Kashinath Bhoosnurmath

**Affiliations:** Global Director, Programmes Operation Eyesight Universal, Hyderabad, India

**Figure:**
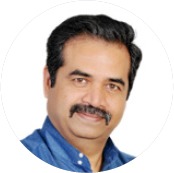
Kashinath Bhoosnurmath

**There is a need for inclusive approach that targets not just the medical causes but also the socio-economic causes of avoidable blindness.**

**Figure:**
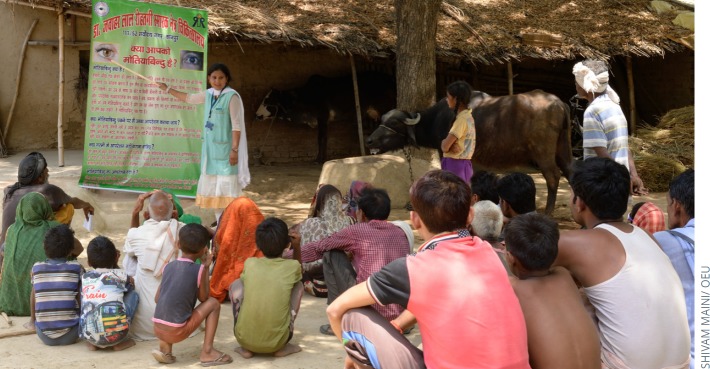
A cataract awareness session. INDIA

The eye health sector shares a uniform approach to eliminating avoidable blindness: making eye care services available to people who would otherwise go unreached. There is a need for an inclusive approach that targets not just the medical causes but also the socio-economic causes of avoidable blindness. Despite significant strides in making services available, avoidable blindness continues to remain a public health issue in many developing countries.

We present a model developed by Operation Eyesight which has proven to be both practical in eliminating avoidable blindness as well as replicable. Our school eye health programme is an integral part of this model and also addresses eye health-related problems of children who have dropped out or have not enrolled in school, children below the age of five and the larger community.

## Hospital-Based Community Eye Health Programme (HBCEHP): The model

### Problem analysis

Poor eye health-seeking behaviour usually stems from inadequate knowledge of eye diseases, harmful cultural beliefs and practices, eye problems viewed as low priority, gender discrimination, lack of affordability and poor mobility amongst the elderly. Often hospitals:

- Provide eye care services but do not empower targeted communities;
- Do not focus on the elimination of avoidable blindness; and
- Make services available free of cost, but poor patients at risk of losing their sight do not access these services.

### Developing the model

The above analyses led us to the conclusion that there were gaps in the services offered by hospitals and that greater effort was needed to empower the target communities.

Through a pilot project implemented from 2009 to 2013 in southern India, we learned that by empowering people living in the service area of the hospital and improving their eye health-seeking behaviour, while continuing to deliver quality eye care services, hospitals could significantly contribute to the elimination of avoidable blindness on a sustainable basis.

This insight led us to develop and successfully scale up a model, named Hospital-Based Community Eye Health Programme (HBCEHP). This model aims to:

- Clear the backlog of avoidable blindness cases, and thereby eliminate avoidable blindness from the service area of a hospital or vision centre;
- Empower target communities and community health workers so that they can address the incidence of blindness and visual impairment.

The projects based on the model are comprehensive and include strengthening hospitals to ensure delivery of quality services; strengthening primary health services, including primary eye care services; and empowering target communities to take ownership and responsibility for their eye health needs. The target communities include school children in the service area of the hospital or vision centre.

## Key components of our HBCEHP model

1. Assess the quantity and quality of services currently being delivered by the hospital.
2. Develop an action plan for implementation of the HBCEHP and improving services.

## Target area selection and cluster formation

The target area is the immediate service area of a secondary eye care centre or vision centre. We delineate the target area into clusters by identifying surrounding villages in such a way that any village can be reached within two hours from the most centrally-located village in the cluster. Each cluster has a population of 5,000 to 25,000 people.

## Recruitment of community health workers or volunteers and other staff

For each cluster, we recruit two community health workers/ volunteers who are part of the target community and live within the cluster. They are usually part of the existing public health system, preferably female, with a minimum qualification of secondary school. Other staff include a project coordinator and a data entry operator.

## Training community health workers or volunteers and other staff

The community health workers or volunteers and other staff undergo a training programme spread over 10 to 20 days. Training is conducted by trained staff from the hospital, based on a curriculum developed by Operation Eyesight.

The training programme focuses on:

- Diseases of the eye, measurement of visual acuity, and classification of blind and visually impaired persons;
- Door-to-door survey methodology and a Knowledge, Attitude, Practice (KAP) survey;
- Formulation of cluster-based micro plans;
- Eye care, maternal and child health care, and immunisation;
- Screening programme methodology, social marketing, referral system and follow-up;
- Health promotion and women's empowerment;
- Monitoring and reporting;
- Other health topics relevant to the target communities.

## Door-to-door surveys

Teams comprising two trained community health workers or volunteers conduct door-to-door surveys in their respective clusters for the entire cluster area using a standard format. The survey lasts two to five months, depending on the population of the area. The survey focuses on identifying people who are blind or visually impaired, with special emphasis on identifying those with cataract, trachoma and refractive errors; assessing people's knowledge, attitude and practice when it comes to eye health (KAP survey); and assessing the immunisation and antenatal/postnatal care status of the population. Validation of the survey is done on a periodic basis by qualified ophthalmic personnel, and the validated data is computerised by a data entry operator.

**Figure 1:**
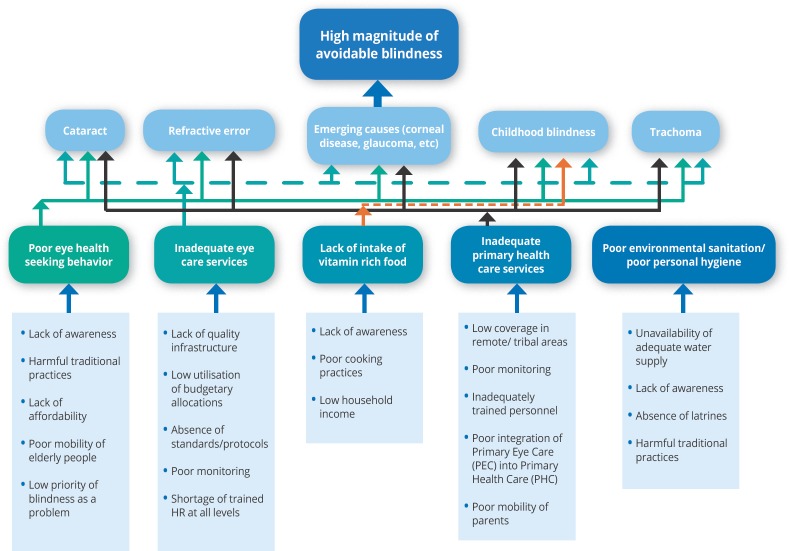
Operation Eyesight Problem Tree.

## Cluster-based annual action plans

Each community health worker or volunteer is assisted to develop a cluster-based annual action plan. This is based on the results of the door-to-door survey and tailored to meet the specific needs of the target community. These plans serve as the basis for all the work undertaken by the workers/ volunteers and ultimately contribute to achievement of the project's specific objectives. A project problem tree guides the development of these plans (Figure 1).

## Community eye care

The following activities are undertaken mainly by the community health workers or volunteers with support from the base hospitals:

- Screening programmes: eye check-ups, treatment for minor ailments and referral to the vision centre and/ or base hospital for appropriate care.
- Implementation of social marketing strategies to encourage target communities to access eye care services being provided by the hospitals.
- Implementation of health promotion and education activities to increase eye care awareness.
- Training of self-help groups in eye care.
- Creation of village vision committees to help identify and refer patients and ensure those needing eye care services access treatment.
- Community-based rehabilitation for those with incurable blindness or visual impairment or other disabilities.

## Primary health care

We work with relevant community-based organisations, NGOs and government departments to implement maternal and child health care activities with a special focus on immunisation services and maternal clinics for antenatal and postnatal care. We also work with these partners to promote primary and non-formal education.

## Hospital care

Community health workers or volunteers and ophthalmic staff running vision centres ensure that all those who require further diagnosis and care present themselves at the base hospitals for surgery or other medical treatment. The staff in the field ensure 100 per cent follow-up.

## Monitoring and reporting

Continuous monitoring of all activities is done by the project coordinator on a daily basis, and by the hospital management on a weekly basis. The results of door-to-door surveys and cluster-based implementation plans serve as the basis for monthly and quarterly monitoring by the hospital management. There are over 30 different registers maintained by field staff. These registers are reviewed regularly, and necessary measures are taken to ensure projects stay on track.

**Figure:**
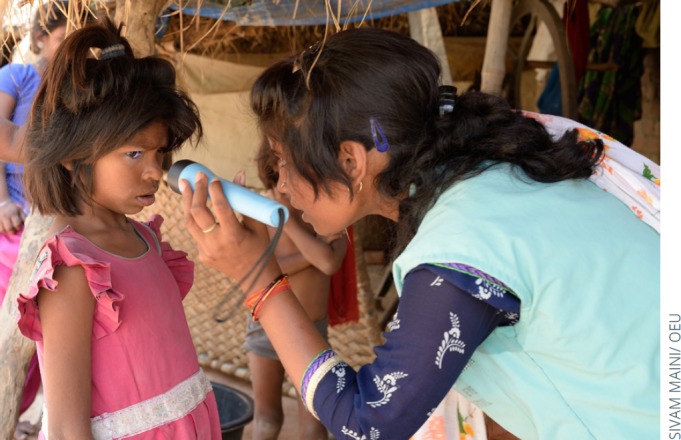
Screening in progress. INDIA

## Declaration of avoidable blindness-free villages/communities

Everyone, regardless of gender, age, religion or abilty to pay, is treated for curable eye conditions. We have a tested methodology based on which we declare villages/ communities as ‘avoidable blindness-free.’ This methodology includes the following key components:

- Verification of cleared of backlog cases, identified through a door-to-door survey;
- A post-project door-to-door survey conducted by community health workers or volunteers under the supervision of an ophthalmologist;
- Treatment of remaining cases and follow-up;
- Certification from local government authorities and ophthalmologists of patients who cannot be treated due to medical or other reasons; and
- A post-project KAP survey to assess the community's current level of eye health-seeking behaviour.

If the results are satisfactory, we declare the village/community as avoidable blindness-free during a public event, which is typically attended by district authorities, elected representatives and other agencies operating in the area.

## Sustainability

The community health workers or volunteers together with the primary eye care/vision centres (which are linked vertically to secondary hospitals) ensure sustained delivery of appropriate care to target populations beyond the project's duration. Community-based action groups, such as village vision committees, women's groups and youth groups, are trained and encouraged to work with community health workers to increase community participation and ensure the project reaches as many people as possible. We also network with relevant government programmes and public health departments to ensure they will provide the required support to hospitals after the projects have ended.

No permanent positions are created for implementation of the projects. In addition, all recurring expenses related to surgical consumables, spectacles, etc. are absorbed by the hospitals as part of their regular annual plans and budgets.

**Table 1:** A snapshot of school children covered under Operation Eyesight's HBCEHP in India

Details	2014	2015	2016
Number of schools covered	279	261	217
Number of teachers trained	335	313	97
Number of children screened	21,647	18,463	15,101
Number of children prescribed spectacles	414	383	229
Number of children provided with spectacles	375	349	211
Number of children identified with other eye diseases (conjunctivitis, other infections, etc.) and referred to a hospital	1,086	1,114	954
Number of children operated on through the project	71	103	126
Number of children identified with Vitamin A deficiency during school screening and provided with Vitamin A supplementation	211	76	3
**Children below five years**			
Number of children who received immunisation as a result of the project	11,665	11,822	37,011

## Results

- Eighty-five per cent of the primary eye care centres are financially sustainable.
- About 70 per cent of the patients who participate in screening programmes and receive treatment are those who were identified during a door-to-door survey and attended health promotion events conducted by community health workers. The remaining 30 per cent of identified patients are counselled at their homes by community health workers, village leaders, etc., and they too eventually undergo treatment (Table 1).
- Surgical conversion rates range between 75 and 92 per cent, and spectacle conversion rates are over 90 per cent.
- Participating hospitals have seen an increase of up to 55 per cent in direct walk-ins from the project areas as compared to pre-project days.

## Conclusion

Operation Eyesight has developed a model that is effective in eliminating avoidable blindness on a sustainable basis, benefiting children and adults alike. By targeting the root causes of avoidable blindness and tailoring our projects to the specific needs of the community, we are able to provide much-needed services that lead to improved eye health and general health for children both within and outside the school system. After successfully piloting our HBCEHP in India, we have expanded our model to other areas of South Asia and Africa where we will continue to prevent blindness and restore sight for more individuals, families and communities.

## Acknowledgements

The author wishes to thank Operation Eyesight India team, especially Mr. Franklin Daniel, Head of Programmes, who is successfully leading the implementation of the model community eye health programme.

